# Report of the Stevens Commission

**Published:** 1890-03

**Authors:** 


### REPORT OF THE STEVENS COMMISSION.

EVER since Dr. Stevens presented his views on the relation of ocular disturbances to functional nervous diseases, especially to chorea and epilepsy, the profession has waited and hoped for their verification.

We doubt if there is any more obstinate disease of pure nervous origin, or one which more frequently baffles us, than does epilepsy. No more important claim has been made in neurological medicine than that of Dr. Stevens, when he said :

> Of sixty-four consecutive cases of well-marked epilepsy in private practice, of which, in every instance, the disease had been of more than one, and in most of many years' duration, and in all of which the treatment has been directed to ocular conditions, medicines having been, except in a single instance, discontinued, thirty-two have remained free from attacks for a time, varying from several months to several years,—a time which, in all ordinary conditions, would enable us to regard the cases cured.

Twenty-one cases were much relieved, and in eleven cases there was no improvement. Here was a report of fifty per cent. of cases of epilepsy cured. What a prospect for hopeless cases! Considering the claim made, it was right that the New York Neurological Society should appoint a committee to inquire into the subject.

The committee was composed of Drs. Seguin, Birdsall, Webster, Moore, Foster, Dana, and Starr. There could hardly have been selected seven men more peculiarly fitted to investigate such a subject. After thirty months of investigation, during which time twenty-eight cases were sent to Dr. Stevens, the committee presents its report. Within the first four months, fourteen cases were, for different reasons, withdrawn, thus leaving fourteen cases available for conclusions. Of these fourteen, five were chorea ; of these, three were improved, two were unimproved ; of the nine cases of epilepsy, two were improved, six were unimproved ; one result, unknown ; no cures out of the fourteen. We must confess that this report is a disappointment, as we had hoped for a better result. But the investigation seems to us to have been fairly conducted by able men, and we do not feel like going behind the returns.

The committee concludes the report by saying :

> In view of these facts, your committee cannot but express the opinion that so far as this investigation has warranted a conclusion, the method of Dr. Stevens does not afford a sufficient degree of relief to patients suffering from chorea and epilepsy, to warrant its adoption or recommendation to the members of the Neurological Society, as a means of cure or as a sole therapeutic measure.

This report naturally does not satisfy Dr. Stevens, and in his reply he complains of bias and unfairness, and adduces the statement of other physicians to show that the result of cases was more favorable than the commission would acknowledge. We must remember, however, that these other observers were not of the commission, and have not watched the cases in every detail, and that Dr. Ranney, at any rate, is already committed as a firm believer in this theory and practice. While we regret that this procedure does not do all that was claimed for it, we are confident that a limited number of cases will be found to depend on these ocular defects.

The entire subject, including correspondence, report and reply, is contained in the November number of *The Journal of Mental and Nervous Diseases*, and constitutes a valuable addition to medical literature.

